# Renal function and risk of dementia: a Mendelian randomization study

**DOI:** 10.1080/0886022X.2024.2411856

**Published:** 2024-10-16

**Authors:** Haowen Huang, Yuan Ren, Jun Wang, Zhiqin Zhang, Jie Zhou, Sansi Chang, Yuelin Zhang, Jun Xue

**Affiliations:** a Division of Nephrology, Huashan Hospital, Fudan University, Shanghai, China; b Shanghai Medical College of Fudan University, Shanghai, China; c Department of Otorhinolaryngology, Head and Neck Surgery, The First Affiliated Hospital, Jiangxi Medical College, Nanchang University, Nanchang, China

**Keywords:** Mendelian randomization, genome-wide association study (GWAS), single nucleotide polymorphism (SNP), renal function, Alzheimer’s disease (AD)

## Abstract

**Background:**

The burgeoning recognition of the nexus between renal functionality and the prevalence of dementia has precipitated a surge in research endeavors. This study aims to substantiate the causal relationship between kidney functionality and dementia.

**Methods:**

We utilized clinical renal function metrics from the Chronic Kidney Disease Genetics (CKDGen) Consortium and diverse dementia types (Alzheimer’s disease [AD] and vascular dementia) from the FinnGen Biobank by using Mendelian randomization analysis. At the stratum of genetic susceptibility, we tested the causal relationship between variations index in renal function and the occurrence of dementia. Inverse-variance weighted (IVW) method was the main analysis, and several supplementary analyses and sensitivity analyses were performed to test the causal estimates.

**Results:**

The findings indicate a significant correlation between each unit increase in cystatin C-based estimated glomerular filtration rate (eGFR-cys) levels was significantly associated with a reduction in the incidence of late-onset Alzheimer’s disease (LOAD) (IVW: OR = 0.35, 95% CI: 0.13–0.91, *p* = 0.031). After adjusting for creatinine-based eGFR (eGFR-cre) and urinary albumin-to-creatinine ratio (UACR), a causal relationship was still identified between elevated levels of eGFR-cys and decreased risk of LOAD (IVW: OR: 0.08; 95% CI: 0.01–0.97, *p* = 0.047). Sensitivity tests demonstrated the reliability of causal estimates.

**Conclusions:**

The association between renal function based on cystatin C and the augmented risk of developing AD lends support to the perspective that regular monitoring of cystatin C may be a valuable investigative biomarker.

## Introduction

Dementia is one of the most common neurodegenerative diseases and the leading cause of disability in people over the age of 65 worldwide [1,2]. It leads to memory loss, thinking difficulties, and reduced ability to carry out daily activities. The most prevalent forms of dementia are Alzheimer’s disease (AD), vascular dementia (VaD), and dementia with Lewy bodies. These dementias differ significantly in etiology, pathomechanisms, symptoms, and progression. Even within the broader classification of AD, early-onset AD (EOAD) and late-onset AD (LOAD) differ in age of onset, genetic factors, pathological features, and symptom progression [3]. It is a major challenge for healthcare professionals and family members [4]. Available data show that AD accounts for approximately 60–80% of all dementia cases and VaD for 20–30% [5–7]. Notably, the number of dementia cases worldwide is expected to triple, reaching 150 million by 2050 [5]. The economic and social burden associated with AD is substantial, yet definitive and effective treatments for the disease continue to be elusive. Research indicates that early identification and targeted management of modifiable risk factors hold promise for slowing the progression of AD and significantly improving the prognosis of dementia [4, 8,9]. It is therefore imperative that these risk factors are effectively detected and intervened upon to facilitate the management and prevention of dementia.

To date, most preventive interventions have primarily targeted factors related to the nervous system, such as skull fractures and concussions [10]. However, these interventions may have inadvertently overlooked the broader population encompassing various physiological states. Notably, cognitive impairment frequently manifests in hemodialysis patients with reduced kidney function, suggesting potential interactions between damaged kidney function and cerebral dysfunction [11]. Chronic kidney dysfunction is widely prevalent globally; however, it is frequently underdiagnosed in its early stages due to the kidney’s efficient compensatory mechanisms. Nevertheless, declining kidney function can lead to systemic implications, affecting various bodily systems, including the nervous, digestive, and circulatory systems [12]. Epidemiological studies have demonstrated a higher prevalence of cognitive impairment among individuals with reduced kidney function when compared to the general population [13]. Observational studies suggest that impaired kidney function may be a significant risk factor for dementia. A 6-year follow-up study further indicates that the impact of impaired kidney function on dementia risk surpasses that of genetic factors [14]. Multiple community-based cohort studies conducted among older adults have further demonstrated that declining estimated glomerular filtration rate (eGFR) is associated with an increased risk of incident AD, allowing for the anticipation of AD onset by more than 1.5 years [15–19]. Furthermore, the urinary albumin/creatinine ratio (UACR) is associated with cognitive dysfunction, including impairments in visual memory and processing speed [20, 21]. However, these studies have limitations including inconsistent measurements of renal function, the bias originating from unobserved and uncontrolled confounding factors (e.g., preexisting vascular disease, depression, APOE ε4), and insufficient statistical robustness due to small population size. Furthermore, observational studies conducted on elderly populations in Japan and normal individuals in Denmark have found no association between reduced glomerular filtration rate and the risk of dementia [16, 22, 23]. Therefore, it remains uncertain whether reduced kidney function plays a causal role in dementia.

Mendelian randomization (MR) is an approach using genetic variants strongly associated with an exposure as potentially unconfounded instruments to elucidate a causal relationship between the exposure and the outcome. Analogous to the principle of randomization, MR analysis is established by Mendel’s law of segregation and independent assortment, effectively mitigating biases stemming from confounding and reverse causation. In this study, we employed MR methods, which are applicable to summarized data from genome-wide association studies (GWASs) to explore the causal association between impaired kidney function and dementia. We selected the most common clinical renal function tests, including creatinine-based estimated glomerular filtration rate (eGFR-cre), cystatin C-based estimated glomerular filtration rate (eGFR-cys), and UACR, as renal function indices of interest, and selected single-nucleotide polymorphisms (SNPs) associated with these indices as instrumental variables to assess whether impaired renal function causally affects the risk of dementia.

## Methods and materials

### Study design

**Figure 1.**
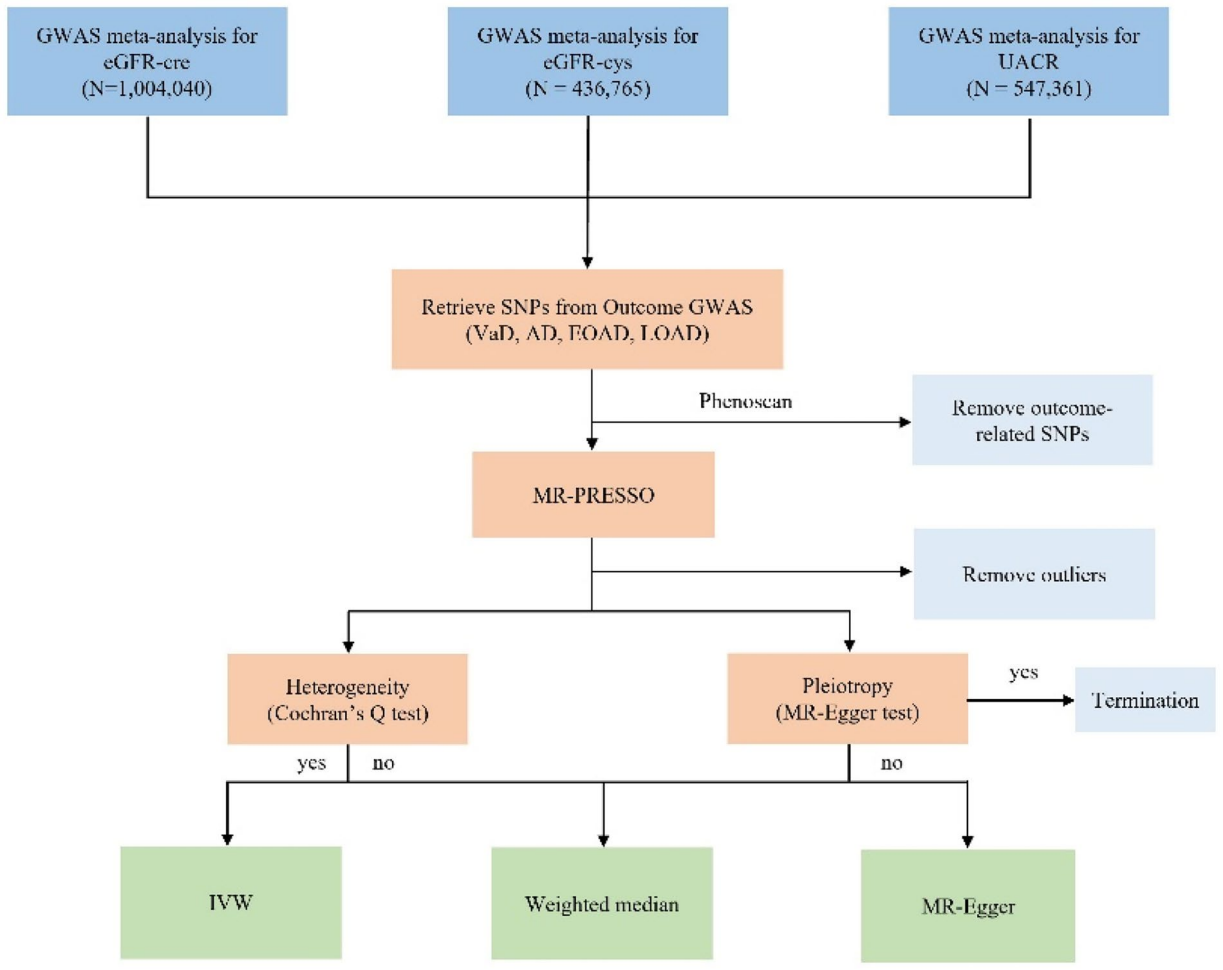
Flow chart of the Mendelian randomization analysis. eGFR-cre, estimated glomerular filtration rate based on creatinine; eGFR-cys, estimated glomerular filtration rate based on serum cystatin C; UACR, urine albumin-to-creatinine ratio; SNP: single-nucleotide polymorphism; VaD, vascular dementia; AD, Alzheimer’s disease (wide definition), EOAD, early-onset Alzheimer’s disease, LOAD, late-onset Alzheimer’s disease; MR: Mendelian randomization; IVW: inverse variance weighted.

To investigate the relationship between renal function and various dementias, a screening of the GWASs of the CKDGen Consortium was conducted, resulting in the selection of eGFR-cre, eGFR-cys, and UACR as exposure indicators (Figure 1) [24, 25]. We also select different dementias in the FinnGen Biobank GWAS for including VaD and AD. To further elucidate the relationship between renal function and the diverse subclasses of AD and to further explore the relationship between renal function and different subclasses of AD. We chose to include AD (wide), EOAD, and LOAD for analysis (Table 1) [26]. As these data were sourced from public databases, no additional ethical approval was required.

**Table 1.** Details of data sources included in the study.

Phenotype	Data source	Year	Sample size	Ancestry
**Exposure**				
eGFR_cre	CKDgen	2021	1,004,040	European
eGFR_cys	CKDgen	2021	436,765	European
UACR	CKDgen	2019	547,361	European
**Outcome**				
VaD	FinnGen Biobank	2023	34,513	European
AD	FinnGen Biobank	2023	309,154	European
EOAD	FinnGen Biobank	2023	303,760	European
LOAD	FinnGen Biobank	2023	307,112	European

eGFR-cre, estimated glomerular filtration rate based on creatinine; eGFR-cys, estimated glomerular filtration rate based on serum cystatin C; UACR, urine albumin-to-creatinine ratio; VaD, vascular dementia, AD, Alzheimer’s disease (wide), EOAD, early-onset Alzheimer’s disease, LOAD, late-onset Alzheimer’s disease; CKD, chronic kidney disease.

### MR analyses

Using common various kidney function-related indicators as exposures and different types of dementia as the outcome, we conducted an MR analysis. Firstly, we extracted SNPs with *p* < 5 × 10^−8^ from the exposure dataset and excluded highly correlated variants with *R*^2^ < 0.001 within a range of 10 Mb to address linkage disequilibrium [27]. In addition, we calculated the F statistic to assess the correlation between the instrumental variables (IVs) and the exposure [28]. F statistic $\left(F=\;\frac{R^{2}}{1-R^{2}}\times \frac{N-k-1}{k}\right)$ greater than 10 was considered indicative of a strong enough correlation to minimize bias resulting from weak IVs. Recognizing the influence of creatinine metabolism on specific genetic loci of eGFR-cre, we eliminated SNPs unrelated to blood urea nitrogen (BUN) (*p* > 0.05) to mitigate interference from other potential confounding factors affecting renal function [29]. To further mitigate potential interference from pleiotropic effects, we employed the MR pleiotropy residual sum and outlier (MR-PRESSO) method, in conjunction with the PhenoScanner database (http://www.phenoscanner.medschl.cam.ac.uk/), for the identification and removal of potential pleiotropic SNPs [30]. Finally, we harmonized the selected exposure and outcome datasets, retaining only SNPs that were unrelated to the outcome (*p* > 5 × 10^–8^). Inverse variance weighting (IVW), MR Egger, and weighted median (WM) methods are selected to evaluate the effect of SNPs representing exposure on the outcome. IVW, as the primary method in MR analysis, combines the Wald ratios for each SNPs with the outcome to obtain overall causal estimate. In addition, MR Egger and the WM provide more flexible estimates for MR analysis.

### Sensitivity analyses

The heterogeneity between the estimated values of each SNP was assessed using Cochran’s Q statistic. If no significant heterogeneity was detected (*p* > 0.05), we employed the fixed-effects IVW model for our MR analysis. In cases where heterogeneity was observed, we utilized the random-effects IVW model [31]. Subsequently, we conducted the MR-Egger intercept test to evaluate the presence of horizontal pleiotropy among the SNPs. A *p* value below 0.05 was considered indicative of potentially unreliable MR results due to horizontal pleiotropy [32]. Furthermore, we conducted a leave-one-out sensitivity analysis by sequentially excluding each SNP to assess the stability of the effect size and identify individual SNPs that disproportionately influenced the association [33].

### Statistical analyses

All statistical analyses were performed using the TwoSampleMR in R (version 4.1.2) [34], and statistical significance in both univariate MR and multivariable MR analysis set at a two-tailed *p* value <0.05.

## Results

### SNPs selection

We selected the latest GWAS associated with kidney function from the CKDgen database, filtering out SNPs with linkage disequilibrium and retained only those SNPs with an F-statistic greater than 10. These SNPs were used as instrumental variables to examine the effect of renal function measures on dementia. After comparing these selected SNPs with the PhenoScanner database, we excluded some SNPs (rs10224002, rs1133415, rs1214761, and rs1458038) because they were associated with potential confounding factors such as ‘diabetes’, ‘hypertension’, and ‘body mass index’ (**Table S1**). Lastly, we utilized MR-PRESSO to exclude any potential pleiotropy (**Table S2**), which ultimately yielded the final set of IVs for the MR analysis.

### Univariate MR analysis

**Figure 2.**
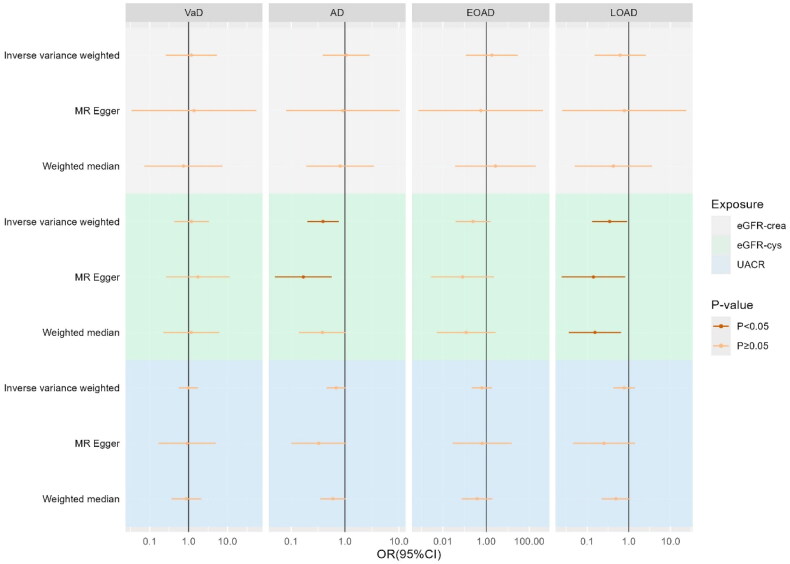
Forest plot of the Univariate MR analyses between renal function and various types of AD. eGFR-cre, estimated glomerular filtration rate based on creatinine; eGFR-cys, estimated glomerular filtration rate based on serum cystatin C; UACR, urine albumin-to-creatinine ratio; SNP: single-nucleotide polymorphism; VaD, vascular dementia; AD, Alzheimer’s disease, EOAD, early-onset Alzheimer’s disease, LOAD, late-onset Alzheimer’s disease; MR: Mendelian randomization.

This study demonstrated a significant association between increased levels of genetically predicted eGFR-cys and reduced risk of AD and LOAD **(**Figure 2**)**. Specifically, our findings suggest a substantial relationship between each unit increase in eGFR-cys levels and lower risk of AD (OR = 0.39, 95% CI: 0.20–0.77, *p* = 0.006) and LOAD (OR = 0.35, 95% CI: 0.13–0.91, *p* = 0.031). Importantly, the results derived from the MR analysis (including MR-Egger and WM methods) were consistent with those derived from the IVW test. Of note, there were no statistically significant associations between eGFR-cre, UACR, and various other forms of dementia.

### Multivariable MR analysis

**Figure 3.**
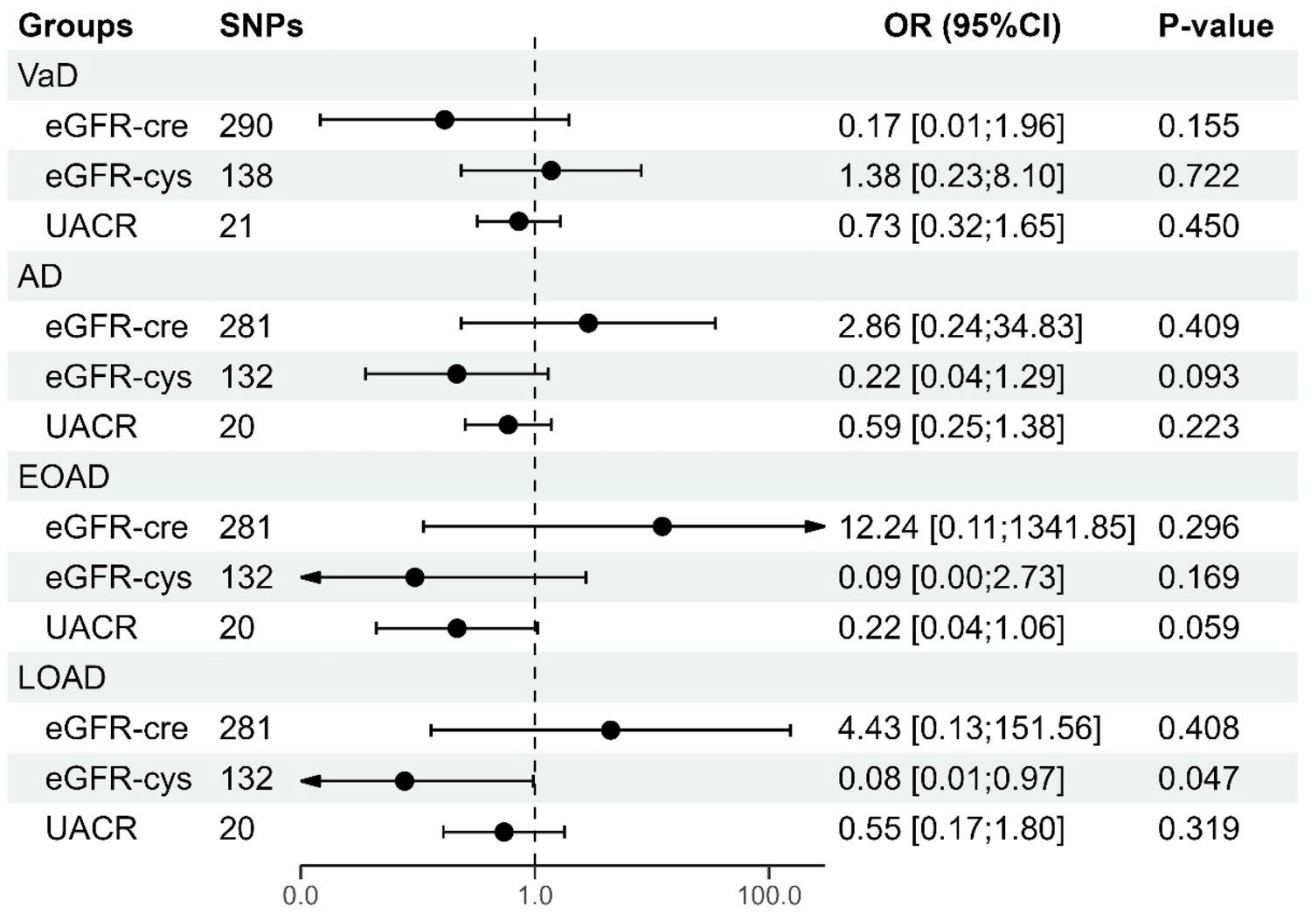
Forest plot which visualizes the multivariable MR analyses between renal function and various types of AD. eGFR-cre, estimated glomerular filtration rate based on creatinine; eGFR-cys, estimated glomerular filtration rate based on serum cystatin C; UACR, urine albumin-to-creatinine ratio; SNP: single-nucleotide polymorphism; VaD, vascular dementia; AD, Alzheimer’s disease, EOAD, early-onset Alzheimer’s disease, LOAD, late-onset Alzheimer’s disease.

To sort out the intricate interactions between renal function exposures, we performed multivariate magnetic resonance (MVMR) analyses to provide a comprehensive assessment of their association with different types of dementia. After adjusting for eGFR-cre and UACR, each unit increase in eGFR-cys was associated with a decreased risk of and LOAD (OR: 0.08; [95% CI: 0.01–0.97], *p* = 0.047) remained statistically significant. However, the causal relationship between rising eGFR-cys and decreased AD risk (OR: 0.22; [95% CI: 0.04–1.29], *p* = 0.093) lost statistical significance **(**Figure 3**)**.

### Sensitivity analysis

A comprehensive sensitivity analysis was conducted on these SNPs after matching the GWAS data representing exposure with the outcome IVs. We assessed the robustness of results and identified the appropriate MR method using MR-Egger intercept, leave-one-out, and the Cochran’s Q heterogeneity test. The MR-Egger intercept tests consistently yielded *p* values > 0.05, indicating the absence of horizontal pleiotropy (Table 2). Furthermore, the leave-one-out analysis demonstrated that the effect of exposure on the outcome was not driven by the effect of a single SNP **(Figure S1-S4)**. Besides, as there was no significant heterogeneity observed (all *p*-values of Cochran’s *Q* > 0.05) (Table 2), we employed the fixed-effects IVW method as the primary approach for our MR analysis.

**Table 2.** Two-sample MR estimates of associations between kidney function biomarkers and dementia using various methods (*p* < 5 × 10^−8^).

Exposure	Outcome	Methods	SNPs	**OR** ^a^	95% CI	**Association** ***p* value**	Cochran’s Q statistic	**Heterogeneity** ***p* value**	**MR-Egger intercept** **(*p* value)**b
eGFR-cre	VaD	IVW	179	1.18	0.26–5.35	0.832	182.2813	0.3973	
	VaD	MR Egger	179	1.37	0.03–54.94	0.868			−4.8e-04 (0.930)
	VaD	WM	179	0.74	0.07–7.42	0.794			
eGFR-cys	VaD	IVW	140	1.19	0.42–3.32	0.746	145.3348	0.3394	
	VaD	MR Egger	140	1.73	0.26–11.4	0.568			−2.2e-03 (0.638)
	VaD	WM	140	1.17	0.22–6.15	0.855			
UACR	VaD	IVW	37	0.99	0.56–1.75	0.968	36.26019	0.4565	
	VaD	MR Egger	37	0.91	0.17–4.99	0.917			1.4e-03 (0.922)
	VaD	WM	37	0.87	0.36–2.1	0.762			
eGFR-cre	AD	IVW	175	1.05	0.39–2.86	0.922	200.0605	0.0856	
	AD	MR Egger	175	0.92	0.08–10.34	0.944			4.4e-04 (0.903)
	AD	WM	175	0.81	0.19–3.47	0.782			
eGFR-cys	AD	IVW	133	0.39	0.20–0.77	0.006	149.8722	0.1369	
	AD	MR Egger	133	0.17	0.05–0.57	0.005			5.0e-03 (0.106)
	AD	WM	133	0.38	0.14–1.03	0.058			
UACR	AD	IVW	33	0.69	0.45–1.03	0.072	41.48905	0.1215	
	AD	MR Egger	33	0.33	0.1–1.07	0.073			1.3e-02 (0.200)
	AD	WM	33	0.6	0.35–1.02	0.06			
eGFR-cre	EOAD	IVW	175	1.87	0.12–29.16	0.654	163.0658	0.7133	
	EOAD	MR Egger	175	0.56	0–433.31	0.866			3.8e-03 (0.698)
	EOAD	WM	175	2.73	0.04–201.13	0.647			
eGFR-cys	EOAD	IVW	133	0.25	0.04–1.61	0.145	118.3041	0.7975	
	EOAD	MR Egger	133	0.08	0–2.34	0.146			6.7e-03 (0.432)
	EOAD	WM	133	0.12	0.01–2.74	0.184			
UACR	EOAD	IVW	34	0.64	0.22–1.88	0.42	34.32425	0.4041	
	EOAD	MR Egger	34	0.66	0.03–15.55	0.799			−4.6e-04 (0.986)
	EOAD	WM	34	0.39	0.07–1.99	0.255			
eGFR-cre	LOAD	IVW	175	0.62	0.15–2.55	0.508	197.7794	0.1045	
	LOAD	MR Egger	175	0.78	0.03–24.06	0.887			−7.3e-04 (0.886)
	LOAD	WM	175	0.43	0.05–3.64	0.435			
eGFR-cys	LOAD	IVW	133	0.35	0.13–0.91	0.031	152.9785	0.1022	
	LOAD	MR Egger	133	0.14	0.02–0.82	0.031			5.3e-03 (0.234)
	LOAD	WM	133	0.15	0.04–0.65	0.011			
UACR	LOAD	IVW	34	0.77	0.43–1.4	0.395	45.45102	0.0730	
	LOAD	MR Egger	34	0.25	0.05–1.42	0.128			2.0e-02 (0.187)
	LOAD	WM	34	0.49	0.22–1.07	0.074			

^a^Estimates represent the effect of a one unit increase in natural log-transformed eGFR-cre, eGFR-cys and UACR as well as that of a one unit decrease in natural log-transformed chemerin. ^b^*p* value of the intercept from the MR-Egger regression analysis. Abbreviations: MR: Mendelian randomization; SNP: single-nucleotide polymorphism; OR: odds ratio; IVW: inverse variance weighted; WM: weighted median; eGFR-cre: creatinine-based estimated glomerular filtration rate; eGFR-cys: cystatin C-based estimated glomerular filtration rate; UACR: urinary albumin-to-creatinine ratio; VaD, vascular dementia; AD, Alzheimer’s disease, EOAD, early-onset Alzheimer’s disease, LOAD, late-onset Alzheimer’s disease; CKD, chronic kidney disease. Bold number indicates that the estimates reach statistical significance.

## Discussion

In the present MR study, we have investigated the causal relationship between genetic predictors of kidney function and dementia. Both univariable and multivariable MR analyses consistently demonstrate that kidney dysfunction, specifically a decline in eGFR-cys, is an independent risk factor for the development of LOAD. Due to the rising prevalence of CKD, it is essential to conduct early screening and risk assessment for dementia in individuals with impaired kidney function, particularly those with significantly elevated levels of cystatin C.

Our study has produced findings in alignment with previous investigations into the association between renal dysfunction and dementia. For instance, a cohort study involving 1,412 elderly participants from the Shanghai Aging Study (SAS) discovered that the combined use of serum creatinine and serum cystatin C, as opposed to the use of creatinine alone, provided more precise risk prediction for all-cause incident AD [10]. Likewise, in a cohort study of 6,256 community-dwelling adults, Stocker et al. found that reduced eGFR based on cystatin C measurement was associated with increased levels of AD-related blood biomarkers, after adjusting for age and sex [35]. However, it is noteworthy that some findings diverged from prior evidence. In a study encompassing 1,562 Japanese elderly subjects, lower eGFR levels determined by serum creatinine concentration displayed no association with AD [22, 23]. Similarly, there was no link between reduced eGFR based on creatinine and the risk of dementia in an observational study conducted within the Copenhagen General Population [16]. It is essential to recognize that the validity of these observational studies may be influenced by confounding variables and reverse causality. Consequently, further research is imperative to establish the causal relationship between kidney function and the risk of dementia.

Vascular factors and neurotoxins may play a key role in the development of dementia. It is well known that chronic renal failure leads to vascular dysfunction, including endothelial damage, amyloid deposition, and microcirculatory changes, all of which are associated with cognitive decline [36,37]. However, in this study, comprehensive MR analysis revealed only a negative association between eGFR-cys and the development of Alzheimer’s disease. Although eGFR-cre is more commonly used clinically to represent renal function, no strong association with dementia was found in this study. We speculate as follows: 1. Cystatin C rises earlier than creatinine in the early stages of renal impairment. It has been shown that cystatin C is more closely correlated with GFR and can rise significantly in mildly reduced renal function, when creatinine levels may still be in the normal range. Thus, the genetic variant of eGFR-cre may be unlikely to represent the integrated impaired renal function, reducing its diagnostic power for dementia [38,39]. 2. Cystatin C is produced by nucleated cells and its metabolism *in vivo* is more constant than that of creatinine. Creatinine correlates with muscle mass, which is significantly correlated with a number of factors including diet, gender, and ethnicity of the person tested, making eGFR-cys a more accurate indicator of changes in renal function [38,39]. 3. Cystatin C’s involvement in the immune process occurs at multiple levels, which plays an important role in immune regulation and apoptosis [40]. The relatively high concentration of cystatin C in the blood may indicate the presence of chronic inflammation, which plays an important role in the pathogenesis of AD [41,42]. The metabolic process of creatinine hardly reflects this chronic inflammatory state of reduced renal function, cystatin C may be a more sensitive predictor of the inflammatory state of AD. In conclusion, these findings suggest that calculating renal function based on serum cystatin C levels may be an early indicator of dementia. However, further in-depth studies on the pathophysiological mechanisms underlying this association are needed.

To the best of our knowledge, this is the first two-sample MR study to confirm a causal relationship between eGFR-cys and AD using summary-level data from a large GWAS, which can avoid potential confounding factors and reverse causation biases inherent in observational studies. Besides, multivariable MR analyses were performed to validate the independent causal effect of eGFR-cys on AD. To ensure the robustness of our findings, we conducted a comprehensive set of sensitivity analyses to scrutinize the MR hypothesis. However, this study has several limitations. Firstly, the GWAS data were limited to individuals of European ancestry, cautioning against direct extrapolation of the causal relationship to other ethnic populations. Secondly, while we identified a causal link between renal functions based on cystatin C and AD, further research is needed to elucidate the underlying mechanisms.

## Conclusions

This is the first study to demonstrate that a decline in eGFR-cys levels is a robust indicator of an elevated risk of AD. Furthermore, it reinforces the notion of a causal link between impaired kidney function and AD. Our investigation, which employed both a univariable two-sample and a multivariable MR approach, yielded a more comprehensive understanding of the potential risk factors that may contribute to the onset and progression of AD. However, the precise pathophysiological mechanisms linking renal dysfunction with AD warrant further investigation to mitigate the risk of future cognitive dysfunction.

## Supplementary Material

Supplementray Tables.xlsx

Supplementary Figures.docx

## Data Availability

The original contributions presented in the study are included in the article/Supplementary Material. All data are publicly available.
