# Supplementary material for: Renal function and risk of dementia: a Mendelian randomization study
Source: Ren Fail. 2024 Oct 16;46(2):2411856. doi: 10.1080/0886022X.2024.2411856 (PMC11485685; doi:10.1080/0886022X.2024.2411856)
Supplement: Supplementary Figures.docx [file IRNF_A_2411856_SM9861.docx]

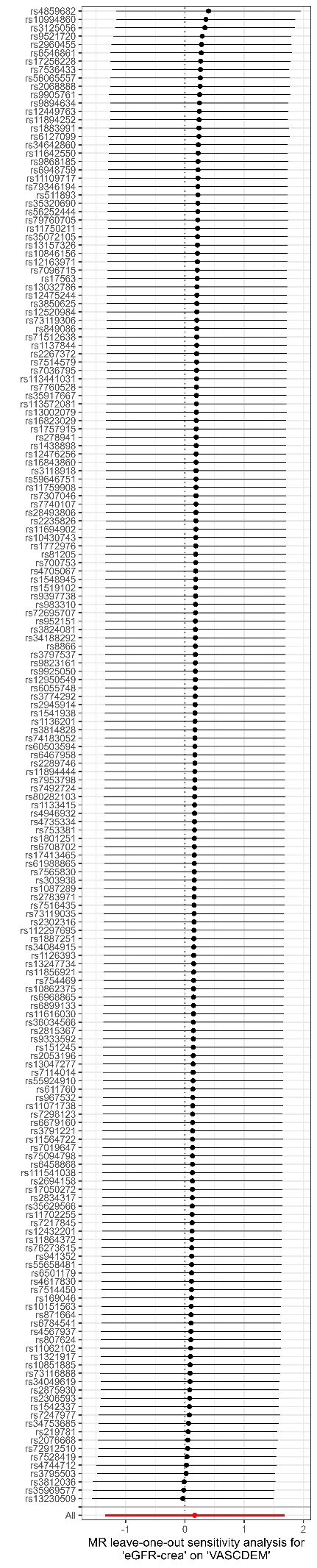

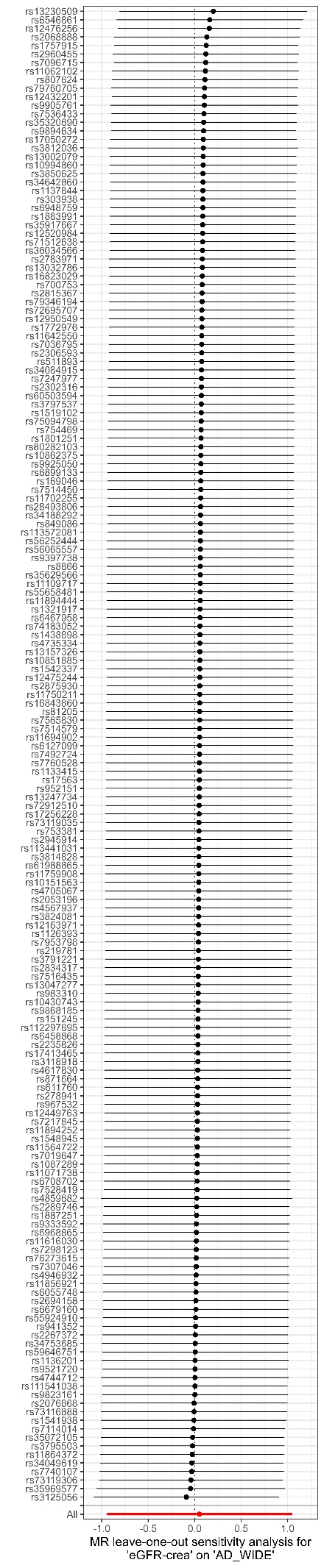


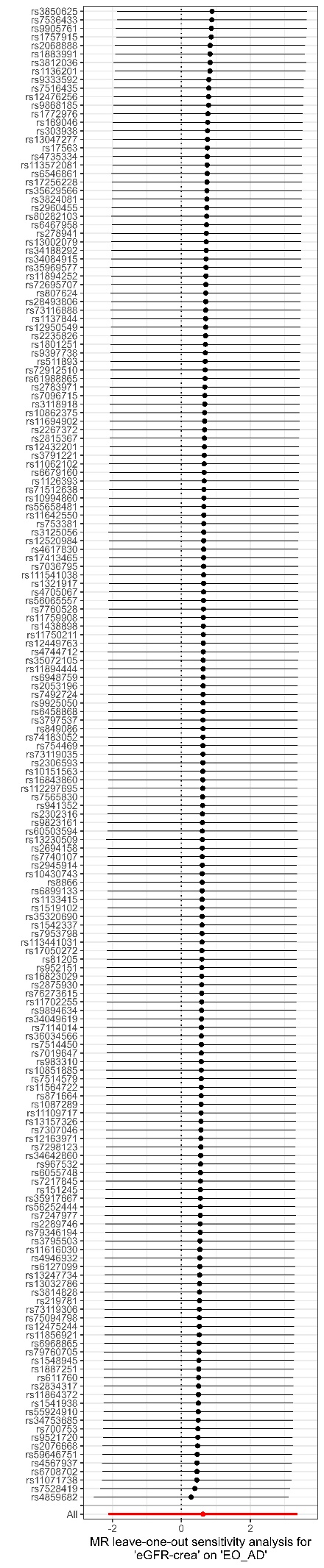

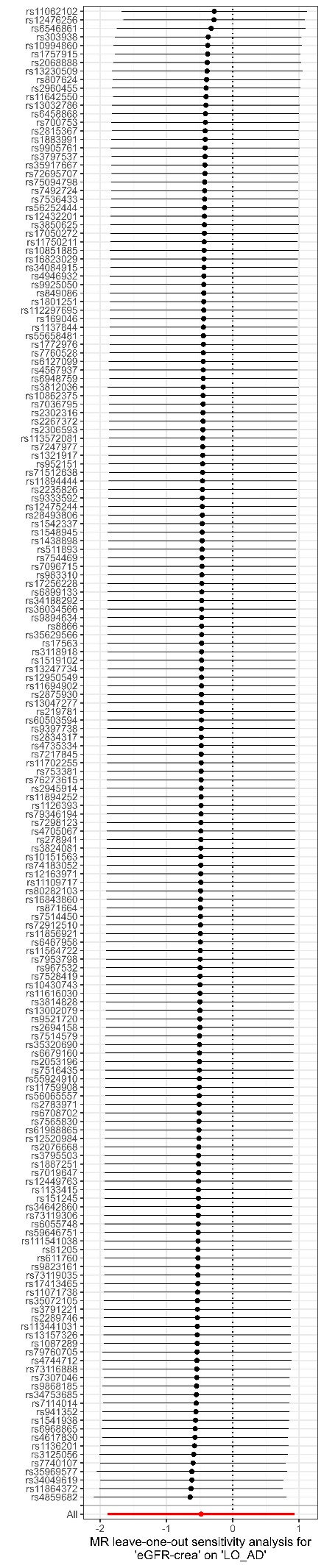


**Figure S1. Leave-one-out plot of the Univariate MR analyses between eGFR-cre and various types of dementia.** eGFR-cre, estimated glomerular filtration rate based on creatinine; VaD, vascular dementia, AD, Alzheimer disease, EOAD, early-onset Alzheimer disease, LOAD, late-onset Alzheimer disease; MR: Mendelian randomization.


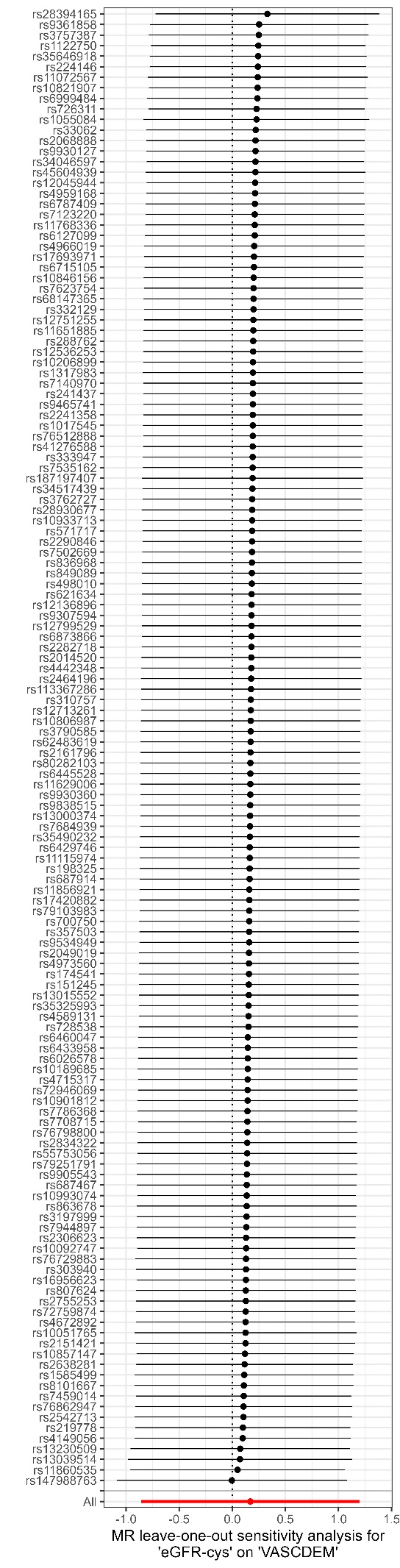

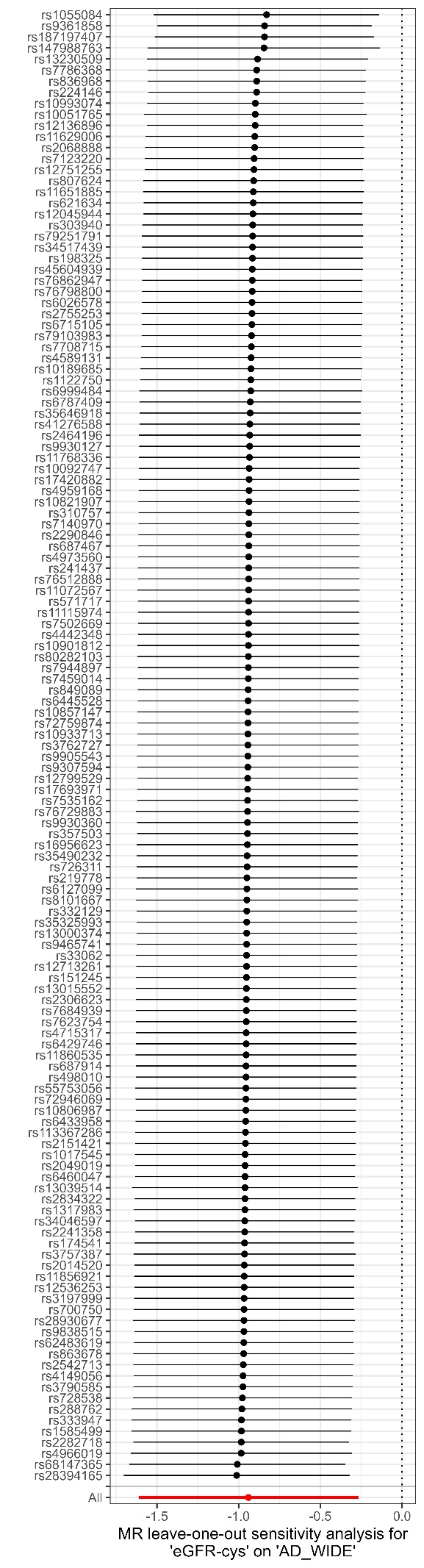


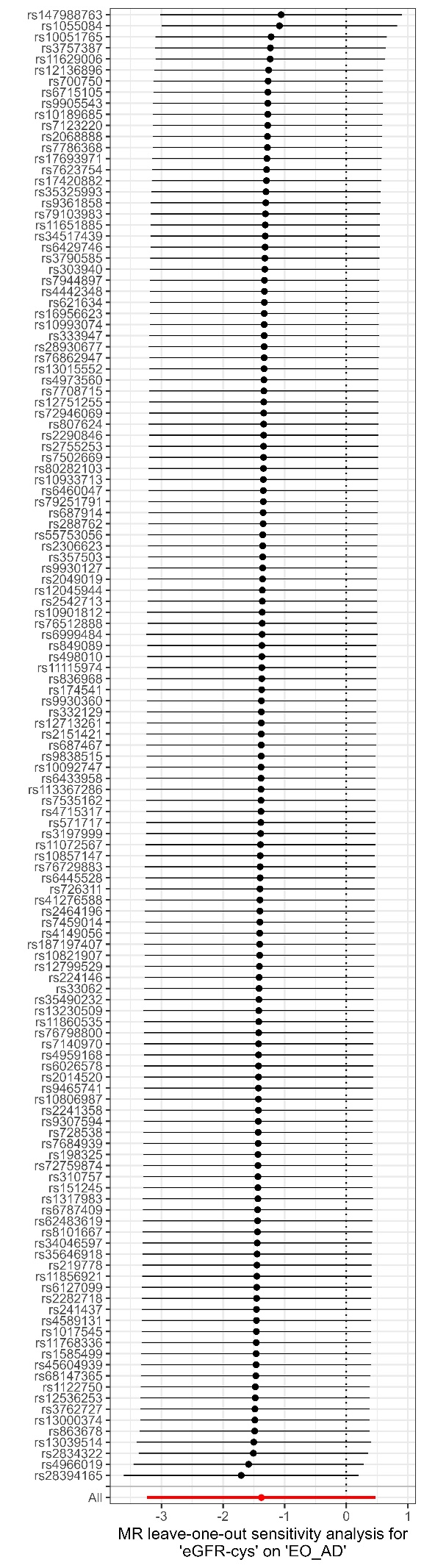

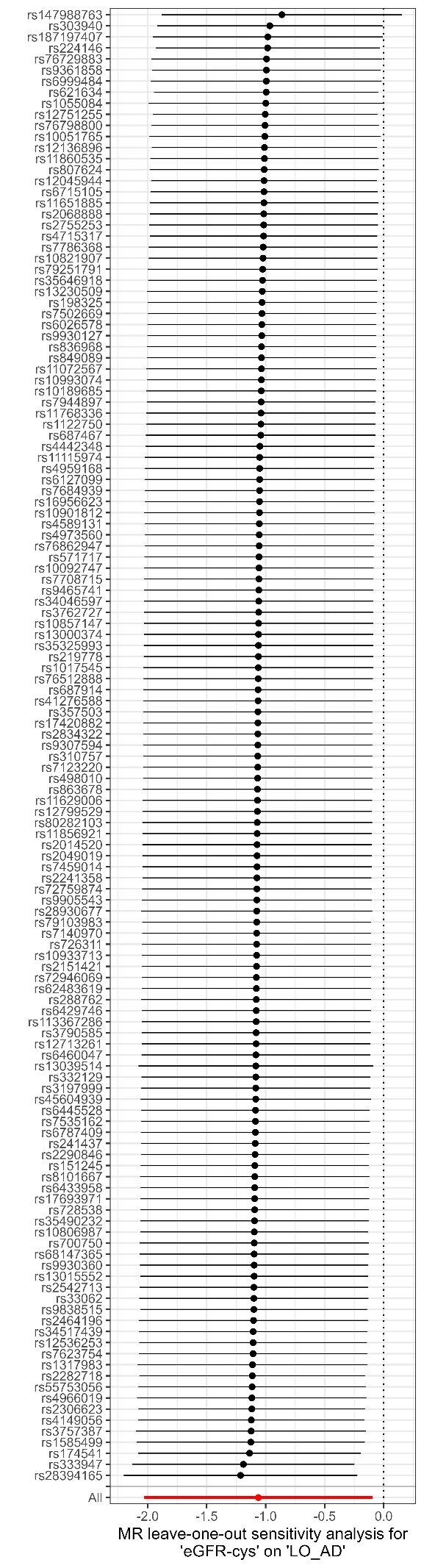


**Figure S2. Leave-one-out plot of the Univariate MR analyses between eGFR-cys and various types of dementia.** eGFR-cys, estimated glomerular filtration rate based on serum cystatin C; VaD, vascular dementia, AD, Alzheimer disease, EOAD, early-onset Alzheimer disease, LOAD, late-onset Alzheimer disease; MR: Mendelian randomization.


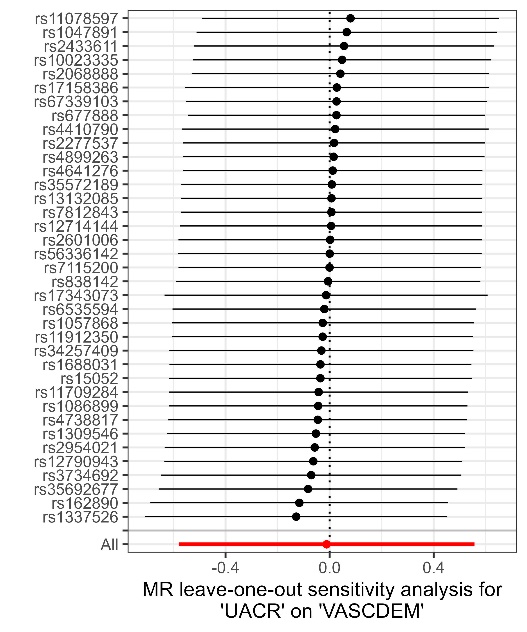

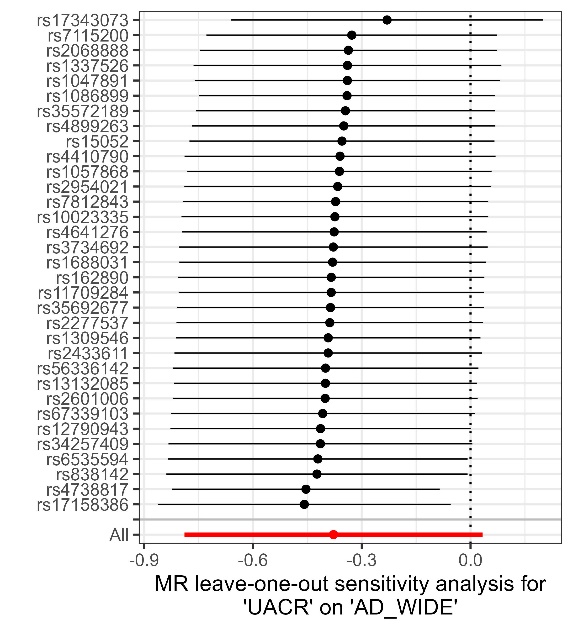


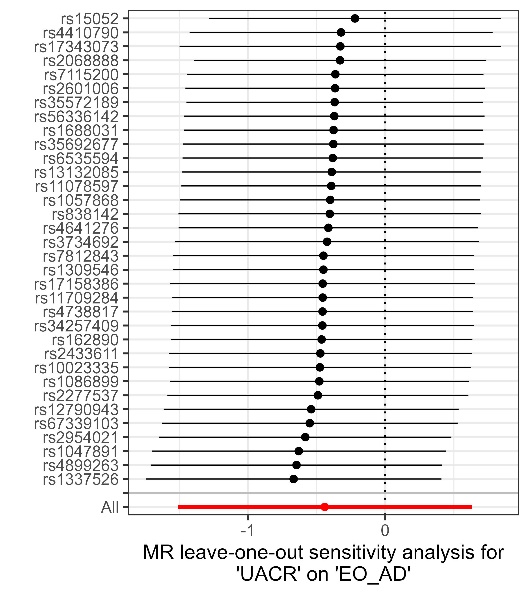

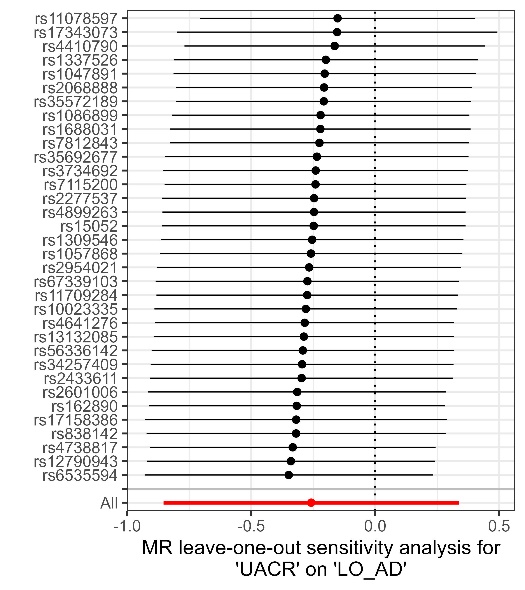


**Figure S3. Leave-one-out plot of the Univariate MR analyses between UACR and various types of dementia.** UACR, urine albumin-to-creatinine ratio; VaD, vascular dementia, AD, Alzheimer disease, EOAD, early-onset Alzheimer disease, LOAD, late-onset Alzheimer disease; MR: Mendelian randomization.
